# Digital quantification of vascular β-amyloid and perivascular plaques in cerebral amyloid angiopathy

**DOI:** 10.21203/rs.3.rs-10550748/v1

**Published:** 2026-08-13

**Authors:** Paniz Mojdeganlou, Jennifer Poirier, Lisa L Barnes, David A Bennett, Sue E Leurgans, Julie A Schneider, Alifiya Kapasi

**Affiliations:** Rush University Medical Center; Rush University Medical Center; Rush University Medical Center; Rush University Medical Center; Rush University Medical Center; Rush University Medical Center; Rush University Medical Center

## Abstract

**Introduction:**

This study aimed to 1) develop a quantitative approach to automate detection of cortical CAA-positive vessels separate from β-amyloid plaques, 2) validate digital pathology classifier for CAA against manual scoring, and 3) characterize the spatial distribution of perivascular β-amyloid plaques in relation to CAA-positive vessels.

**Methods:**

Digital pathology classifiers were trained on the HALO AI platform to distinguish cortical CAA-positive vessels from β-amyloid plaques in the calcarine cortex. Classifiers were applied to digitized histopathologic slides (n = 100), previously rated by expert neuropathologists. Slide level digital quantitative measures for CAA (CAA classified area and vascular β-amyloid percent positivity) and for β-amyloid plaques (β-amyloid plaque classified area) were compared with manual CAA scores and Alzheimer’s disease neuropathologic change (ADNC). Perivascular plaques, defined as β-amyloid plaques within a 0- to 150-micron concentric distance from the center of CAA-positive vessels, were identified.

**Results:**

Participants had a mean age of 91.1 (SD = 5.6) years at death, with 76% being women. Digital pathology quantification for CAA classified area and vascular β-amyloid percent positivity showed strong correlations with semi-quantitative manual CAA scores (r_S_= 0.96 and r_S_ = 0.79, respectively). The number of CAA-positive vessels per slide ranged from 0 to 820, with a median of 3 vessels in score 1, 12 in score 2, 153 in score 3, and 399 in score 4. Separately, digital quantification of β-amyloid plaque showed strong correlations with Thal phase (r = 0.80) and moderate correlations with ADNC (r = 0.60). Interestingly, the total β-amyloid plaque area was relatively similar across manual CAA scores. However, spatial proximity analysis revealed that cases with higher CAA scores had more perivascular β-amyloid plaques close to CAA-positive vessels.

**Conclusion:**

Digital pathology classifiers reliably distinguish cortical CAA vessels from β-amyloid plaques and can be utilized to study plaque proximity to CAA-positive vessels severity, supporting the use of digital pathology to advance understanding of CAA pathogenesis.

## INTRODUCTION

Cerebral amyloid angiopathy (CAA) is considered a small vessel disease characterized by the deposition of β-amyloid (Aβ) protein and often thickening of the walls of cortical and leptomeningeal small vessels [16]. Pathologic lesions including intracerebral hemorrhage, microbleeds, microinfarcts, ischemic brain injury, enlarged perivascular spaces, and white matter hyperintensities are widely considered as markers of disease severity [10]. Further, CAA is frequently observed in the brains of older persons, can co-occur with Alzheimer disease (AD) pathology [9], and has been associated with higher risk of Alzheimer’s dementia and cognitive decline.[8]

Postmortem histopathologic evaluation of brain tissue remains the ‘gold standard’ for the diagnosis and understanding of CAA because it provides direct visualization of vascular β-amyloid within vessels, as well as assessment of vessel and/or parenchymal damage linked with CAA, which cannot yet be fully captured through clinical or imaging techniques alone [32]. Autopsy-based assessments of CAA have typically relied on semi-quantitative evaluation on the extent of CAA severity as mild, moderate, or severe. However, semi-quantitively scales vary across raters and does not provide biological insight. Further, the availability of different and multiple grading schemes for CAA can hinder cross-study comparability [18,25,37]

Digital pathology has emerged as a robust and transformative tool for studying the diverse and complex spectrum of β-amyloid (Aβ) pathologies that can be observed on immunohistochemical-stained slides. By combining whole-slide imaging (WSI), high-resolution scanning, and computational image analysis, digital pathology allows for precise, scalable, and multifaceted investigations of Aβ deposition patterns that were previously limited by manual methods [15,27]. In this study, we utilized machine learning-based classifiers to automate detection and quantification of parenchymal CAA-positive vessels, defined as a vessel with any vascular β-amyloid deposition, and separately β-amyloid plaques in the calcarine cortex. Additionally, we examined the burden of perivascular plaques surrounding CAA-positive vessels.

## METHODS

### Participants

Participants were included from four ongoing longitudinal clinical-pathologic cohort studies of aging: the Religious Orders Study (ROS), the Rush Memory and Aging Project (MAP), the Minority Aging Research Study (MARS), and the Rush Clinical Core (Clinical Core).

ROS, started in 1994, has enrolled Catholic nuns, priests, and brothers from over 40 communities across the United States. MAP has recruited older adults from retirement communities and subsidized senior housing facilities throughout Chicagoland and northeastern Illinois since 1997 [6]. MARS (started in 2004) [3] and Clinical Core (renewed in 2008) [31] recruit older African American adults from the metropolitan Chicago area and surrounding suburbs.

All cohorts enrolled participants without a known clinical diagnosis of dementia at baseline. Each study was approved by the Rush University Medical Center Institutional Review Board. All participants underwent annual clinical evaluations and signed written informed consent along with an Anatomical Gift Act for brain donation, required for ROS and MAP, and optional for MARS and Clinical Core.

#### Clinical assessment

All participants received a standardized clinical evaluation that incorporated a medical history, neurologic examination, and a battery of 19 cognitive tests at baseline and during each annual follow-up visit. First, a neuropsychologist, blinded to participant demographics, reviewed clinical data to rate cognitive impairment. A clinician then evaluated all clinical data, examined the participants, and provided a final diagnosis. At the time of death, a clinician, blinded to postmortem data, reviewed clinical data from all study years to determine the participant’s dementia status proximate to death [7].

The diagnosis of dementia follows consensus guidelines [20], requiring evidence of cognitive decline relative to a person’s previous level of performance with impairment in multiple cognitive domains. Mild cognitive impairment (MCI) was assigned to participants with cognitive impairment determined by a neuropsychologist if the participant did not meet the criteria for dementia [5]. Those without dementia or MCI were classified as having no cognitive impairment (NCI).

#### Diagnostic Neuropathology Assessment

The median postmortem interval was 8.75 hours (IQR = 6.00 to 13.75). Brain autopsies followed a standardized protocol [28,30]. One cerebral hemisphere was cut into 1-cm coronal slabs and frozen at −80°C. The contralateral hemisphere was fixed in 4% paraformaldehyde and similarly slabbed. Tissue blocks were collected, embedded in paraffin, sectioned at 6 μm, and immunostained to evaluate CAA, Alzheimer’s disease neuropathologic changes (ADNC), and other neurodegenerative pathologies.

##### *Pathologic diagnosis of Alzheimer’s disease*:

Diagnosis was assigned according to the National Institute on Aging-Alzheimer’s Association (NIA-AA) criteria [14]. Neuritic plaques and neurofibrillary tangles were manually quantified within a 1-mm^2^ grid in an area of greatest density using modified Bielschowsky silver-stained sections from five brain regions (midfrontal, middle temporal, entorhinal, inferior parietal cortices, and hippocampus) to determine Braak stage and CERAD score [40].Thal phase was determined by reviewing immunohistochemically stained sections of seven brain regions (midfrontal, middle temporal, inferior parietal cortices, hippocampus, basal ganglia, cerebellum, and midbrain) using monoclonal antibodies against Aβ (4G8 1:9000; Bio legend, San Diego, CA) [14].

###### Manual scoring of CAA

CAA was scored by neuropathologists on 4G8-immunostained sections of the calcarine cortex, using a semiquantitative scale from 0 to 4, based on both the extent of β-amyloid accumulation and the number of affected vessels. A score of 0 was assigned when no β-amyloid deposition was observed in the vessel walls. A score of 1 corresponds to partial β-amyloid deposition in one or more vessels. A score of 2 indicated circumferential β-amyloid deposition in one to ten vessels, while a score of 3 was assigned when more than ten vessels exhibited circumferential deposition. Finally, a score of 4 was given when over 75% of vessels demonstrated circumferential β-amyloid involvement [8]. Figure 1 demonstrates histology images representative of the manual CAA scores.

####### *LATE-NC*:

TDP-43 pathology was assessed in eight brain regions: the amygdala, hippocampus (CA1/subiculum and dentate gyrus), entorhinal, midfrontal, middle temporal, anterior temporal tip, inferior orbital frontal cortices. Immunohistochemistry with phosphorylated monoclonal TAR5P-1D3, pS409/410 antibodies (dilution: 1:10,000; Bio legend, CA) was performed on 6-μm sections [22].

Cytoplasmic TDP-43 inclusions were counted in a 0.25 mm2 area of greatest density, and their severity was scored from 0 (no inclusions) to 6 (severe) [22]. LATE-NC staging followed established consensus guidelines, with four levels: stage 0 (no TDP-43 detected), stage 1 (TDP-43 inclusions limited to the amygdala), stage 2 (extension to the hippocampus or entorhinal cortex), and stage 3 (extension into any neocortical region) [1,24]

####### *Lewy bodies*:

Lewy body disease (LBd) pathology was evaluated in 7 brain regions (midfrontal, midtemporal, entorhinal, anterior cingulate and inferior parietal cortices, substantia nigra, and amygdala) using phosphorylated α-synuclein immunostaining (dilution: 1:20,000; Wako chemicals, VA) [29].

#### Digital Scanning and Quality Control

All slides were loaded into the Leica Aperio AT2 console and were digitized at 20x magnification to obtain whole slide images (WSI). WSIs were stored in SVS format and managed through the web-based eSlide Manager database.

Quality control was performed for each WSI to ensure suitability for digital image analysis. This involved careful inspection for slide preparation artifacts, including staining inconsistencies, tissue folds, residual debris, and air bubbles, along with scanning artifacts such as poor focus, streaking artifacts, slide markings, and dust. Identifying and addressing these artifacts is essential to ensure the reliability of downstream image analysis [15].

#### Digital Quantification for CAA-positive vessels and parenchymal β-amyloid plaques

##### *Training and Optimization of Digital Pathology Classifier*:

The HALO AI DenseNet module (Indica Labs), a machine learning–based object detection classifier, was trained to detect three ‘classes’: (1) parenchymal CAA-positive vessels, (2) β-amyloid plaques, and (3) background tissue. For this study, parenchymal CAA-positive vessels were defined as cortical vessels with any vascular β-amyloid deposition. The classifier training dataset comprised approximately 200 manual annotations for parenchymal CAA-positive vessels and 2,000 for parenchymal β-amyloid plaques from 10 4G8-immunostained whole slide images. Model performance was refined through iterative cross-validation and visual inspection. Misclassifications were addressed by adjusting training parameters and refining annotations. To improve detection accuracy, a minimum object area threshold of 400 μm^2^ for CAA-positive vessels and 100 μm^2^ for β-amyloid plaques were implemented. A probability threshold of 0.90 was used for both classifiers to exclude low-confidence detections and minimize false positives.

##### *Vascular β-Amyloid percent Positivity*:

To quantify the intensity of β-amyloid deposition within CAA-positive vessels, the CAA vessel ‘class’ was combined with the area quantification module. This combination created the vascular β-amyloid percent positivity algorithm, which captures the percent positivity of staining in a CAA-positive vessel. For each CAA-positive vessel, optical density thresholds were applied to classify staining into three intensity ranges: weak (yellow pixels), moderate (orange pixels), or strong (red pixels). Thresholds were determined through review by an expert neuropathologist. The algorithm output provides the percentage of vessel wall area stained positive for β-amyloid. Figure 2 demonstrates the workflow for developing classifiers. Figure 3 (panels a and b) demonstrates the classification of CAA–positive vessels and vascular β-amyloid percent positivity. Figure 4 shows the classification of parenchymal β-amyloid plaques.

###### Case Selection for Classifier Validation

To compare the digital classifiers for CAA (both CAA classification and vascular β-amyloid percent positivity) with manual CAA scoring, we selected a stratified random sample of participants (n = 100) with historical CAA scoring assessment (scores 0–4) in the calcarine cortex. Participants were evenly distributed, with 20 participants selected for each CAA score. Slides selected consisted of 100 4G8-immunostained 6μm sections and did not overlap with the 10 immunostained slides used to train the classifier. Analysis regions were manually annotated to include only cortical gray matter. White matter and meningeal vessels were excluded to ensure detection was restricted to grey matter cortex. For each region analyzed, slide-level summary data and individual-vessel level data for both CAA classified area and vascular β-amyloid percent positivity were available (Fig. 2).

### Proximity Analysis

To assess the spatial distribution of β-amyloid plaques relative to CAA-positive vessels, we employed the Proximity Analysis module in the HALO-AI platform. This module was configured to identify parenchymal β-amyloid plaques located within a 150-μm radius of each CAA-positive vessel, divided into three concentric 50-μm intervals (0–50 μm, 50–100 μm, and 100–150 μm). Perivascular plaques defined for this study included deposition of Aβ in the glia limitans as well as parenchymal β-amyloid plaques within a 150-um distance from each CAA-positive vessel detected. The proximity analysis pairs the center of each classified CAA-positive vessel with the center of the nearest classified β-amyloid plaque, enabling quantification of perivascular plaque counts within each distance interval. The proximity analysis provides information on the number of plaques centered within each 50-μm concentric ring intervals; thus, we calculated a perivascular plaque density as the total number of plaques divided by the area of each concentric ring. These metrics allowed us to systematically characterize the relationship between CAA-positive vessels and the surrounding perivascular β-amyloid plaque pathology. Figure 5 (panel a) demonstrates a conceptual illustration of perivascular plaque proximity to a CAA-positive vessel.

#### Software and Hardware Specifications

Image analysis was performed using HALO AI (Indica Labs, version 4.0) with the DenseNet Mask classifier, Area Quantification Module, and Proximity Analysis Module. Training and analysis were conducted on a high-performance HP Z4 G4 Intel XeonW-2265 64 GB RAM workstation equipped with a Nvidia RTX A6000 48GB Graphics Card, running Windows 11.

### Statistical analysis

For each slide, several variables of interest were examined, both at the individual vessel and across the entire cortical region of interest:

### Variables for each vessel or plaque:

#### CAA-positive vessel

Each vessel classified positive for β-amyloid

#### Vascular β-amyloid percent positivity

Percent area of each classified CAA-positive vessel positive for β-amyloid

#### CAA vessel size

Area of each CAA-positive vessel; square-root transformation applied

#### Perivascular plaque density

The total number of parenchymal β-amyloid plaques centered within each concentric ring (0–50-μm; 50–100-μm; 100–150-μm) surrounding each CAA-positive vessel, divided by the area of each concentric ring. *The calculated areas for each concentric ring were 7,850 μm*^2^ *for the 0–50 μm ring, 23,550 μm*^2^ *for the 50–100 μm ring, and 39,250 μm*^2^ *for the 100–150 μm rin*g.

#### Parenchymal β-amyloid plaques

Each parenchymal β-amyloid plaque classified.

### Variables for the entire cortical region of interest:

#### Total CAA-positive vessel count:

*Total n*umber of vessels classified as positive for β-amyloid in the cortex.

##### CAA classified area (mm^2^)

Area of cortex occupied by all classified vessels positive for β-amyloid.

##### Total vascular β-amyloid percent positivity

Percent area of all classified CAA-positive vessels positive for β-amyloid.

##### Total parenchymal β-amyloid plaques

Total number of parenchymal β-amyloid plaques classified in the cortex.

##### Plaque classified area (mm^2^)

Area of cortex occupied by all classified parenchymal β-amyloid plaques.

##### Total perivascular plaque density

The total number of parenchymal β-amyloid plaques divided by each concentric ring surrounding each CAA-positive vessel, averaged across the entire cortex. *Values were multiplied 10*^*4*^

Digital classification for CAA and parenchymal β-amyloid plaques, as well as vascular β-amyloid percent positivity, were first examined for skewness and outliers by calculating skewness coefficients and inspecting quantile–quantile (Q–Q) plots across all slides. Scatterplots and Spearman correlations were used to compare digital data with historical manual pathology scores. We utilized Spearman correlations to evaluate associations between digital metrics for CAA and β-amyloid with semi-quantitative CAA scores, Thal phase, Braak stage, CERAD score, ADNC, TDP-43, and Lewy body pathologies. We assessed demographic associations with CAA using t-tests for sex and Spearman correlations for age at death and years of education. Comparisons between APOE-e4 carriers and non-carriers were also performed using t-tests. One-way ANOVA was used to compare total and perivascular plaque-classified area across manual CAA scores.

All statistical analyses were performed using SAS/STAT version 9.4 (SAS Institute Inc., Cary, NC) on a Hewlett Packard ProLiant ML350 server with LINUX operating system. Statistical significance was defined as a p-value < 0.05.

## RESULTS

A total of 100 participants were included. The demographic, clinical, and pathological characteristics of study participants are presented in Table 1. On average, age at death was 91 (SD = 5.82) years and mean education was 17 (SD = 3.42) years, with 76 Women in the group. The APOE-e4 allele was present in 24 of 91 participants (26%). Clinically, the mean estimated Mini-Mental State Examination (MMSE) score proximate to death was 22 (SD = 7.80). Based on clinical diagnosis proximate to death, 30 participants had no cognitive impairment (NCI), 20 had mild cognitive impairment (MCI), and 50 had dementia.

Alzheimer’s Disease Neuropathologic Change (ADNC) was absent in 6 participants, evaluated as low likelihood in 13, intermediate in 51, and high in 30 participants. In terms of Thal phase (A-score), 6 participants had no β-amyloid pathology in any brain region assessed, 8 had β-amyloid pathology localized to the neocortex and/or hippocampus, 32 had β-amyloid involving the basal ganglia, and 53 had β-amyloid involving the substantia nigra and/or cerebellum. Braak score was I/II in 10 participants, III/IV in 54 participants, and V/VI in 36 participants, with no participants scoring 0. Neuritic plaques were common in the neocortex, with 85 participants having a moderate-to-severe burden.

### Comparing digital quantification of CAA-positive vessels and vascular β-amyloid positivity across manual CAA scores

Application of machine-learning based pathology classifiers facilitated automated detection of CAA-positive vessels (classified CAA area) and quantitative assessment of vascular β-amyloid percent positivity for each individual vessel as well as slide-level assessment (Table 2). The number of CAA-positive vessels detected ranged from 0 to 1,017, with a median of 3 vessels in score 1, 12 in score 2, 153 in score 3, and 399 in score 4 (Table 2). Vascular β-amyloid positivity ranged from 0% to 80.30%, with a median value of 24.9% in score 1, 30% in score 2, 39.7% in score 3, and 57.9% in score 4.

Digital CAA metrics, both CAA-classified area and vascular β-amyloid percent positivity, showed excellent correlations with manual CAA scores (r = 0.94 and r = 0.81, respectively; both p < 0.001). The digital classified CAA area increased in a stepwise manner across higher manual CAA scores, with minimal-to-low CAA classified areas detected in scores 0–2 (none-to-mild) and a pronounced rise at score 3 (moderate) and 4 (severe). We also find that there is a broader range of classified CAA area in cases scored as 4 (severe), with vessel counts ranging from 132 to 1017, likely reflecting the within-grade differences in CAA burden that are not captured by manual ratings. Similarly, digital vascular β-amyloid percent positivity also increased progressively across higher manual CAA grades. However, in contrast to the CAA classified area, vascular β-amyloid percent positivity displayed a more gradual, linear relationship across the full range of manual CAA scores, with higher levels of vascular β-amyloid percent positivity noted even at lower CAA score (Fig. 3, panels c and d).

Separately, we investigated the distribution of vessel size across manual CAA scores. We find that the majority of CAA-positive vessels fell within the 25^th –^ 75th percentile range of vessel size. However, differences emerged at the extremes of distribution. In cases with lower CAA scores, few CAA-positive vessels exceeded a sq-root transformed vessel area of of 50 μm^2^ vessel area (which corresponds to 2,500 μm^2^ vessel area). In contrast, higher CAA scores had a much higher proportion of larger CAA-positive vessels, including vessels exceeding a sq-root transformed vessel area of 100μm^2^, which was seen in the most advanced CAA score (Fig. 6). Vessel size showed moderate-to-strong correlations with CAA-classified area (r = 0.73, p < 0.001) and with vascular β-amyloid percent positivity (r = 0.56, p < 0.001)

#### Associations of digital quantification of CAA-positive vessels with demographics, APOE-e4, clinical status, and neurodegenerative pathologies.

Demographic comparisons revealed no significant sex differences in CAA burden (women: 0.24 ± 0.40 mm^2^; men: 0.16 ± 0.29 mm^2^; t (98) = 0.85, p = 0.40) and no associations with age at death (r = 0.06, p = 0.54) or years of education (r = −0.02, p = 0.88). As expected, APOE-e4 carriers exhibited significantly greater CAA classified area compared to non-carriers (mean = 0.51 ± 0.45 mm^2^ vs. 0.12 ± 0.31 mm^2^; t (89) = − 4.59, p < 0.001). The median number of CAA-positive vessels among APOE-e4 carriers was 269 vs. 6 in noncarriers. Across clinical status, the median number of CAA-positive vessels was higher in those with dementia (median = 65) vs. MCI (med = 12.5 vessels) vs. normal cognition (med = 4.5 vessels). When examining correlation with neuropathologies, we find moderate associations with Thal phase, Braak stage, and CERAD score, and weaker correlations with a pathological diagnosis of Alzheimer’s disease. No associations were seen with LATE-NC or Lewy body pathology (Fig. 7).

#### β-amyloid plaque load and perivascular plaque proximity to CAA-positive vessels.

The median area of β-amyloid plaques was 1.36 mm^2^ (IQR = 0.22 to 3.14). Plaque-classified area in the calcarine cortex correlated strongly with Thal phase (r = 0.74, p < 0.001) and moderately with ADNC stage (r = 0.56, p < 0.001). Next, we examined whether β-amyloid classified plaque area differed across manual CAA scores. Interestingly, there was no significant differences in the total plaque-classified area across manual CAA scores (score 1 to score 4; ANOVA (F_df1,df2_=0.09), p = 0.96); suggesting that total β-amyloid plaque burden remained relatively stable across CAA scores (Fig. 4, panel c).

We next examined whether the distribution of perivascular β-amyloid plaque differed across manual CAA scores. Spatial proximity analyses examined the distribution of β-amyloid plaques around CAA-positive vessels within a 150-μm radius of each CAA-positive vessel, divided into three concentric 50-μm intervals (rings): 0–50 μm, 50–100 μm, and 100–150 μm.

In contrast to the total β-amyloid plaque burden, which on average remained relatively stable across CAA severity scores, the number and density of perivascular plaques was higher in cases with advanced CAA. Within the 150 μm perivascular zone, participants with higher CAA scores exhibited more perivascular plaques, with the highest perivascular plaque density in the innermost 50 μm ring (50–100 μm) (Table 3, Fig. 5, panel b).

## DISCUSSION

In this study, we developed and validated a digital pathology approach for automated detection and quantification of CAA-positive vessels and separately parenchymal β-amyloid plaques in the calcarine cortex, as well as the characterization of the spatial relationship between CAA-positive vessels and β-amyloid perivascular plaques. Digital classification measures derived from HALO analysis demonstrated high accuracy and robustness in detecting CAA-positive vessels, providing objective metrics for both the number of affected vessels and the intensity of vascular β-amyloid deposition. By leveraging this digital pathology workflow, we not only reproduced the manual CAA scores traditionally assigned by neuropathologists, but also extended assessment by generating objective, reproducible metrics, and spatial context at the whole-slide level.

Manual semi-quantitative CAA scores are widely used in research and diagnostic settings, enabling pathologists to identify vascular β-amyloid deposition using traditional light microscopy. However, these conventional approaches are limited by their ordinal nature, compresses biologic variability, and unable to quantify pathology across the whole region of interest or capture spatial relationships between CAA and differing patterns of β-amyloid plaques [8,11,17].

To address these limitations, we applied whole-slide digital quantification, which systematically analyzes each CAA-positive vessel and β-amyloid plaque deposit, enabling objective and comprehensive assessment of vascular and parenchymal β-amyloid pathology across the entire tissue section. We find that while both digital CAA classifiers correlate strongly with manual scores, digital metrics of vascular β-amyloid percent positivity provides a more sensitive readout of early-stage vascular β-amyloid deposition, whereas digital metrics of classified CAA area more distinctly separated none-to-mild from the moderate-to-severe burden cases. Importantly, both the digital metrics for CAA classified area and vascular β-amyloid percent positivity reveals the within score biologic variability, particularly at the advanced CAA stages. Our findings are consistent with other groups utilizing similar machine learning-based workflows to comprehensively study different morphologic types of Aβ deposits [26,36]. Furthermore, these studies confirm the reliability of automated pathology tools in replicating expert evaluations [15]. Together, these findings highlight the potential use of digital pathology and machine learning-based workflows to be able to detect subtle variabilities of vascular β-amyloid burden across-and within-score differences, which would not be captured by manual categorical ratings, and even the possibility to refine CAA phenotyping and enable reproducible cross-study comparisons.

A notable observation in this study was the spatial clustering of perivascular β-amyloid plaques around CAA-positive vessels. Our definition of perivascular plaques included deposition of Aβ in the glia limitans as well as parenchymal β-amyloid plaques within a 150-um distance from each CAA-positive vessel detected by the classifier. While the total parenchymal β-amyloid plaque burden across the entire cortex did not differ across CAA severity groups, cases with higher CAA scores exhibited more perivascular β-amyloid plaques, with a greater plaque density in the innermost 50 μm ring, and lower densities within the outer rings. While the underlying etiology of why perivascular plaques cluster around CAA-positive vessel remains unclear, it is possible that this pattern reflects a shared perivascular pathway of β-amyloid deposition and clearance. Under normal physiological conditions, soluble Aβ is cleared along basement membranes of small arteries and arterioles; however, as CAA accumulates, these drainage pathways become progressively obstructed by vascular Aβ deposits. This impairment in perivascular clearance may lead to the accumulation of Aβ in the adjacent parenchyma, producing a gradient of plaque density that is highest in the immediate perivascular space, which diminishes with increasing distance from the vessel wall [35,39]. Additionally, CAA-affected vessels may serve as local sources of Aβ deposition into the surrounding tissue, promoting perivascular plaque aggregation. Furthermore, localized inflammation associated with CAA could further promote Aβ aggregation and/or reduce clearance efficiency near vessels [34] and therefore reinforcing the spatial clustering of plaques. Taken together, our findings support the hypothesis that vascular dysfunction in the presence of CAA may play a role in shaping how close β-amyloid plaques surround vessels [13,38]

The presence of APOE-e4 allele is the strongest genetic risk factor for late-onset Alzheimer’s disease. Consistent with prior studies from our group and others [23], carriers of the APOE-e4 allele showed a higher CAA burden. Although the present analysis focused on parenchymal arterial/arteriole CAA burden, APOE-e4 has been linked to greater meningeal and capillary CAA [33,41]. APOE-e4 influences several inflammatory and vascular pathways that could exacerbate Aβ deposition. For example, e4-expressing microglia can exhibit proinflammatory gene expression profiles, which may promote vasculature frailty and less efficient clearance of insoluble A-beta deposits. Additionally, arterial stiffening is exacerbated, and the neurovascular unit is compromised among APOE-e4 carriers, both of which in turn impact perivascular drainage [2,4,12,21]. All these pathways are amplified by aging and vascular risk factors. Clarifying how APOE-dependent alterations in vascular integrity, perivascular clearance, and glial signaling converge on vascular β-amyloid plaque accumulation and/or clearance will be important to identify mechanistic therapeutic targets for CAA.

The implementation of digital workflows within neuropathology laboratories and cores has gained substantial momentum in recent years. However, establishing the infrastructure required to support these workflows presents several challenges, including the need for sustained funding, dedicated space, specialized equipment, and collaboration among multidisciplinary teams to enable standardization, integration, and long-term storage of digital data. To maximize the translational potential of these approaches, harmonization of digital pathology practices across multiple sites and cohorts will be essential. Developing shared reference datasets, unified metadata standards, and coordinated quality-control procedures will facilitate cross-site benchmarking, enhance reproducibility, and enable integration of digital metrics into larger, multi-cohort analyses, ultimately advancing collaborative efforts in Alzheimer’s disease and cerebrovascular research [19].

This study had several limitations. The group size, while sufficient for validation, limits the statistical power for subgroup analyses by APOE genotype or clinical diagnosis. The HALO classifier was trained on a relatively large dataset of parenchymal CAA with a minimum size threshold, and while this feature was necessary to minimize false positive detections, it also excluded the evaluation of capillary CAA, a notable pathologic feature implicated in microvascular injury. Furthermore, all digital assessments were from a single brain region (calcarine cortex), which precludes study of regional distribution of CAA. Lastly, because the design of this study only captured CAA-positive vessels, we were unable to assess the proportion of CAA-negative vessels to determine whether perivascular plaques surround CAA-negative vessels. This study also had multiple strengths. First, whole-slide analysis ensured that all available tissue was evaluated, providing a comprehensive assessment and improving reproducibility. Second, integrating spatial analysis of perivascular plaques provided novel insight into how vascular and parenchymal amyloid deposits interact at the microscopic level; an aspect often overlooked in routine scoring systems. Third, validating digital measures against established neuropathologic scores strengthens confidence in accuracy and reliability. Lastly, all slides were manually annotated, eliminating the potential for tissue-related artifacts to interfere with digital metric outputs.

## CONCLUSIONS

In summary, these findings support the use of automated image-analysis pipelines as reliable tools for large-scale neuropathologic studies and for advancing our understanding of how vascular and parenchymal amyloid deposition interact in the aging and Alzheimer’s disease brain.

## Figures and Tables

**Figure 1:**
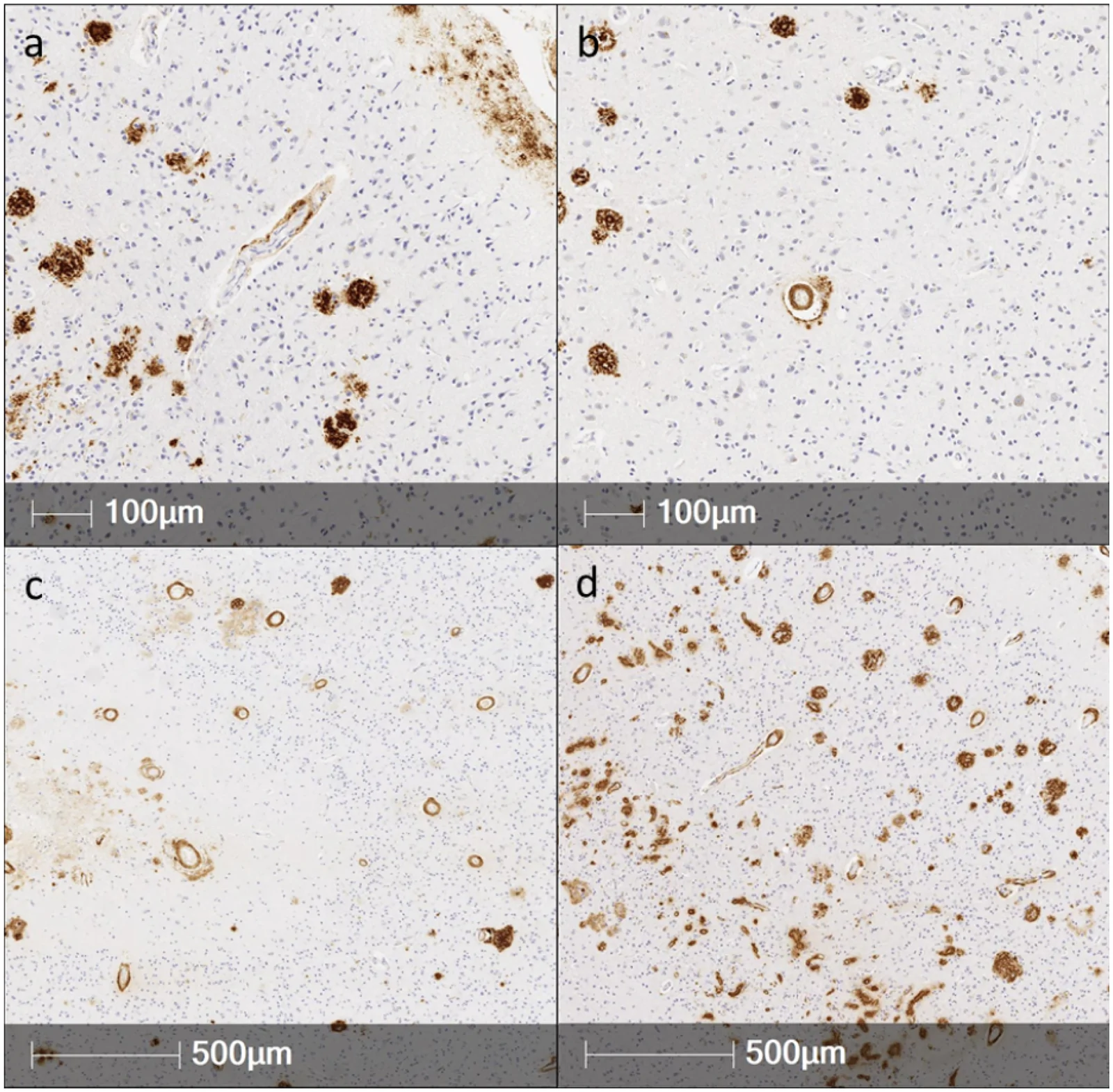
Representative histopathology images for manual CAA scores. Images represent 4G8-immunostained slides demonstrating manual CAA score 1 (a), CAA score 2 (b), CAA score 3 (c), and CAA score 4 (d).

**Figure 2:**
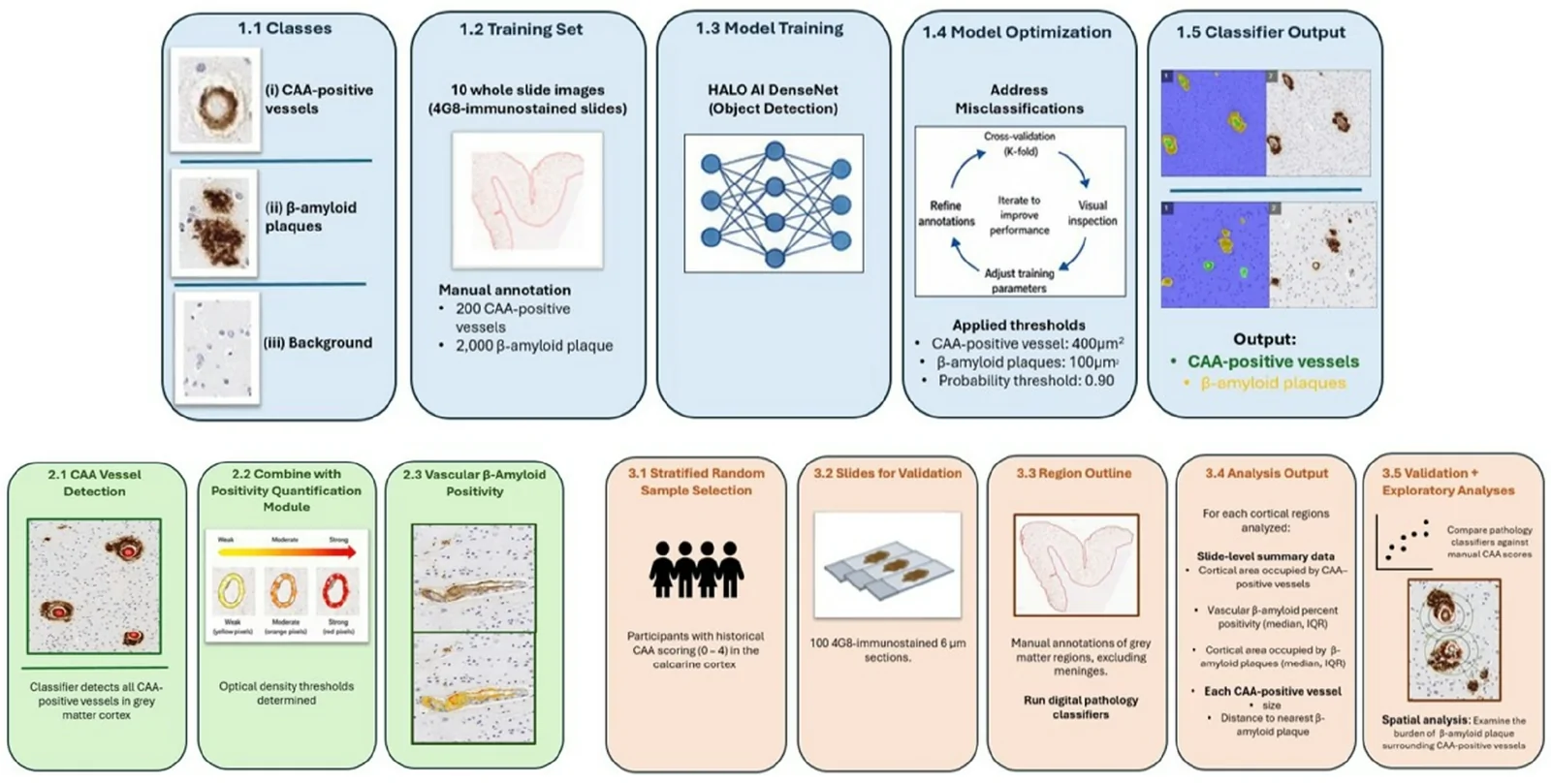
Digital pathology workflow for detecting CAA-positive vessels, vascular β-amyloid percent positivity, and parenchymal β-amyloid plaques.

**Figure 3:**
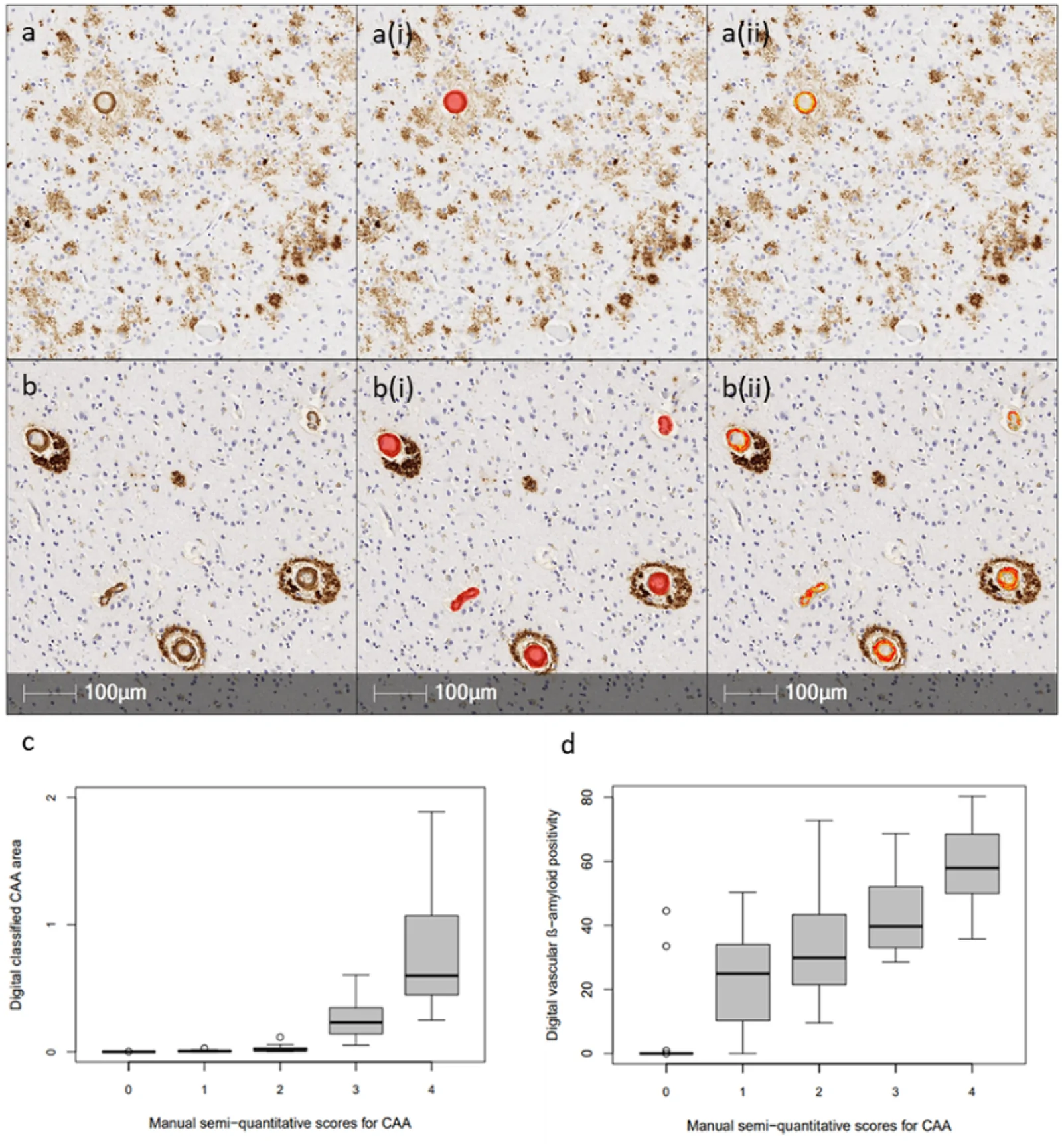
Digital quantification for CAA-positive vessels and vascular β-amyloid percent positivity Panels a, a(i), and a(ii) represent one example from a unique slide of the 4G8-stained image of a CAA vessel, the classifier detection of the CAA-positive vessel and the vascular β-amyloid percent positivity of the same vessel. Panels b, b(i), and b(ii) represent a second example from another unique slide. Boxplots show the digital classified CAA area (panel c) and the vascular β-amyloid percent positivity (panel d) by manual CAA scores.

**Figure 4:**
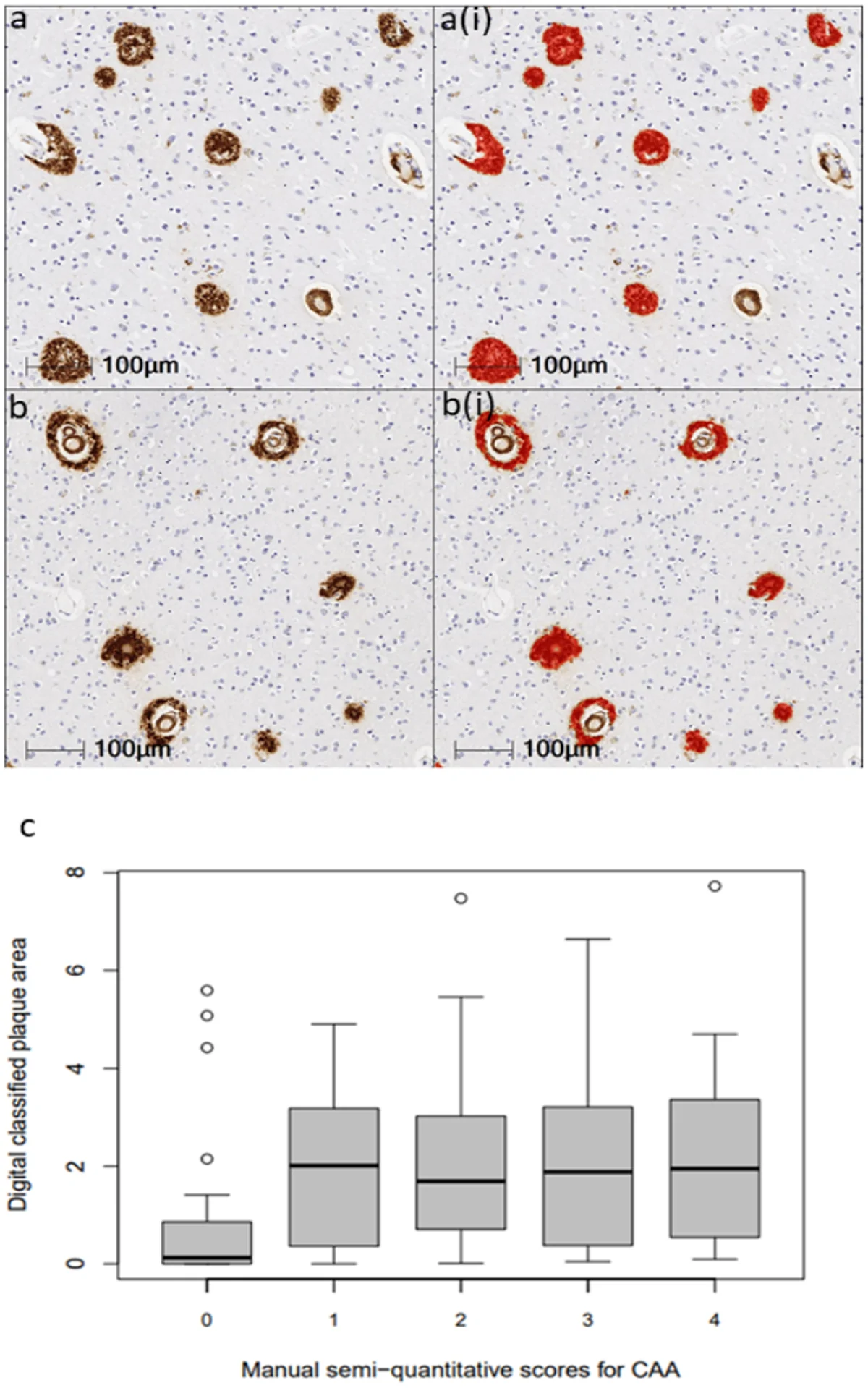
Digital quantification for total parenchymal β-amyloid plaques. Panels a and a(i) represent one example from a unique slide of the 4G8-stained image of parenchymal β-amyloid plaques and the digital markup detection of classified β-amyloid plaques. Panels b and b(i) represent a second example from another unique slide. Boxplot c demonstrates the comparison of total classified plaque area across manual CAA scores.

**Figure 5:**
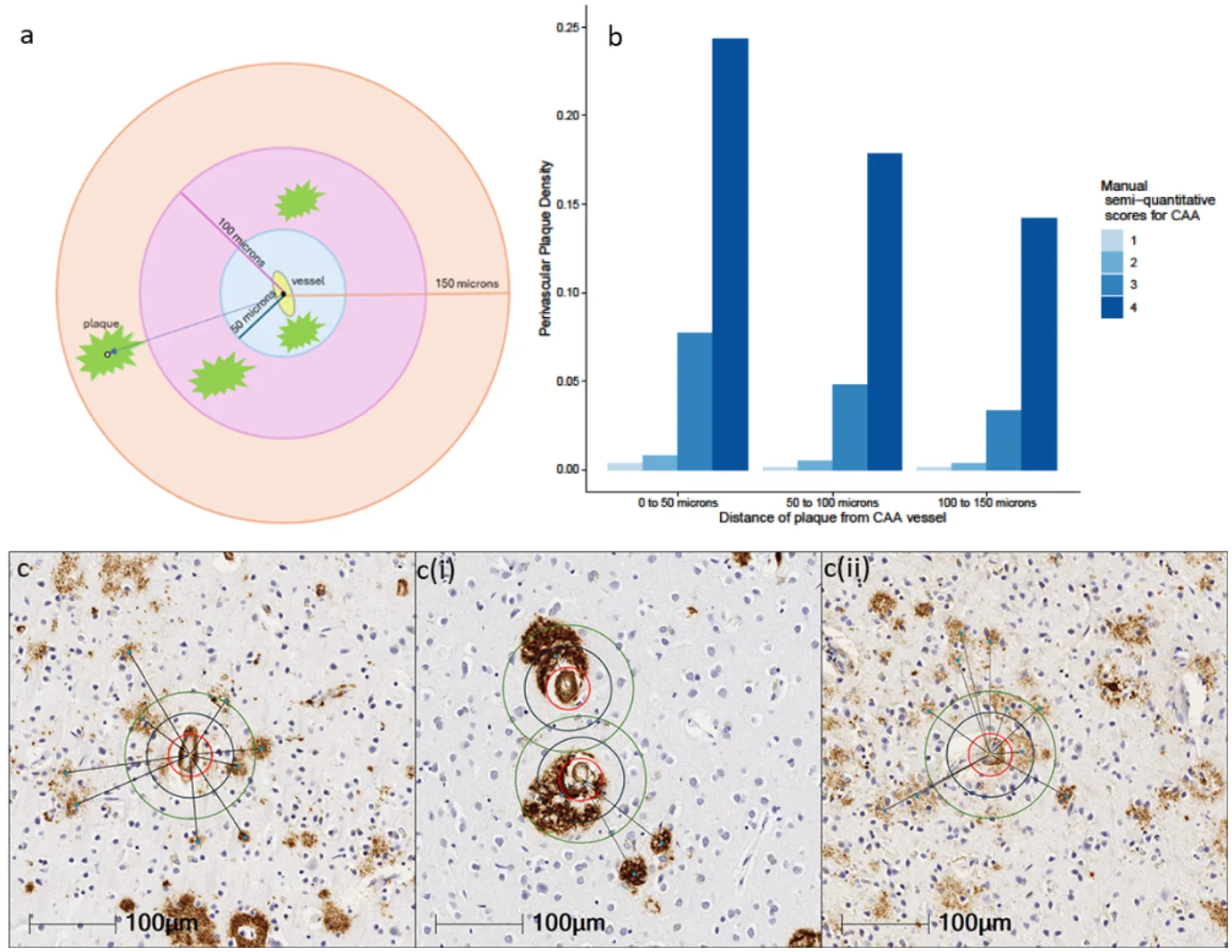
Proximity of perivascular β-amyloid plaques to CAA-positive vessels. Panel (a) illustrates perivascular plaque proximity to CAA-positive vessels. A plaque was classified as perivascular if the center of the plaque was located within a circle with a 150-μm radius of the center of a CAA-positive vessel. Perivascular plaques were further divided according to which three concentric 50-μm rings (0–50 μm, 50–100 μm, and 100–150 μm) contained the center of the plaque. Panel (b) shows a bar plot of the density of β-amyloid perivascular plaques in each concentric ring surrounding CAA-positive vessels by manual CAA scores. Panels (c, ci, and cii) illustrate three separate examples of the detection of perivascular plaques around CAA-positive vessels via HALO proximity analysis module. The proximity analysis pairs the center of each classified CAA-positive vessel with the center of the nearest classified β-amyloid plaque, which can be identified as within the 150μm radius or extending beyond the 150μm radius.

**Figure 6:**
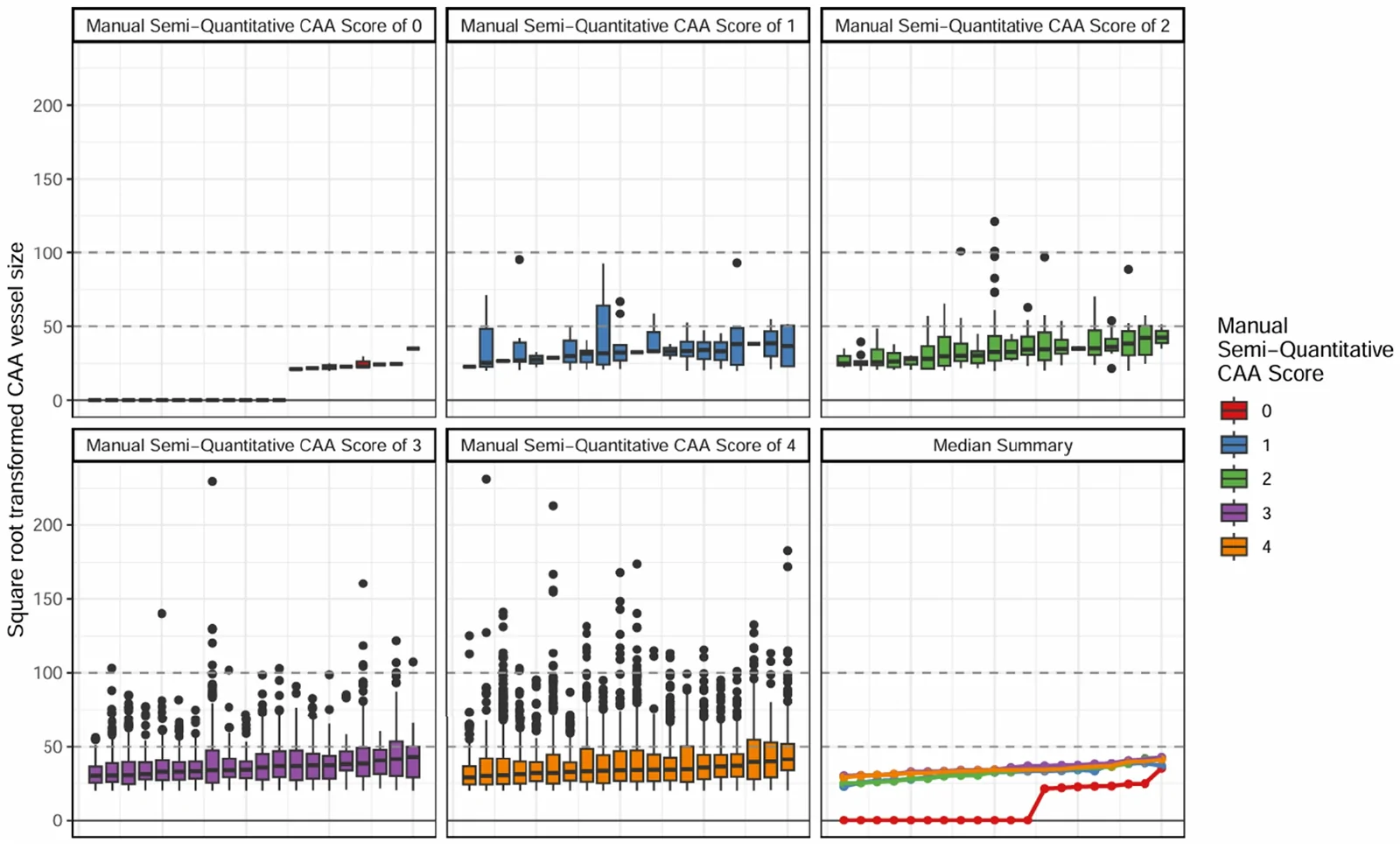
CAA vessel size by manual CAA scores Each boxplot represents the size of the CAA-positive vessels (expressed as square-root μm2) across a single slide. The participants are arranged in increasing order of median size within manual CAA-Score.

**Figure 7:**
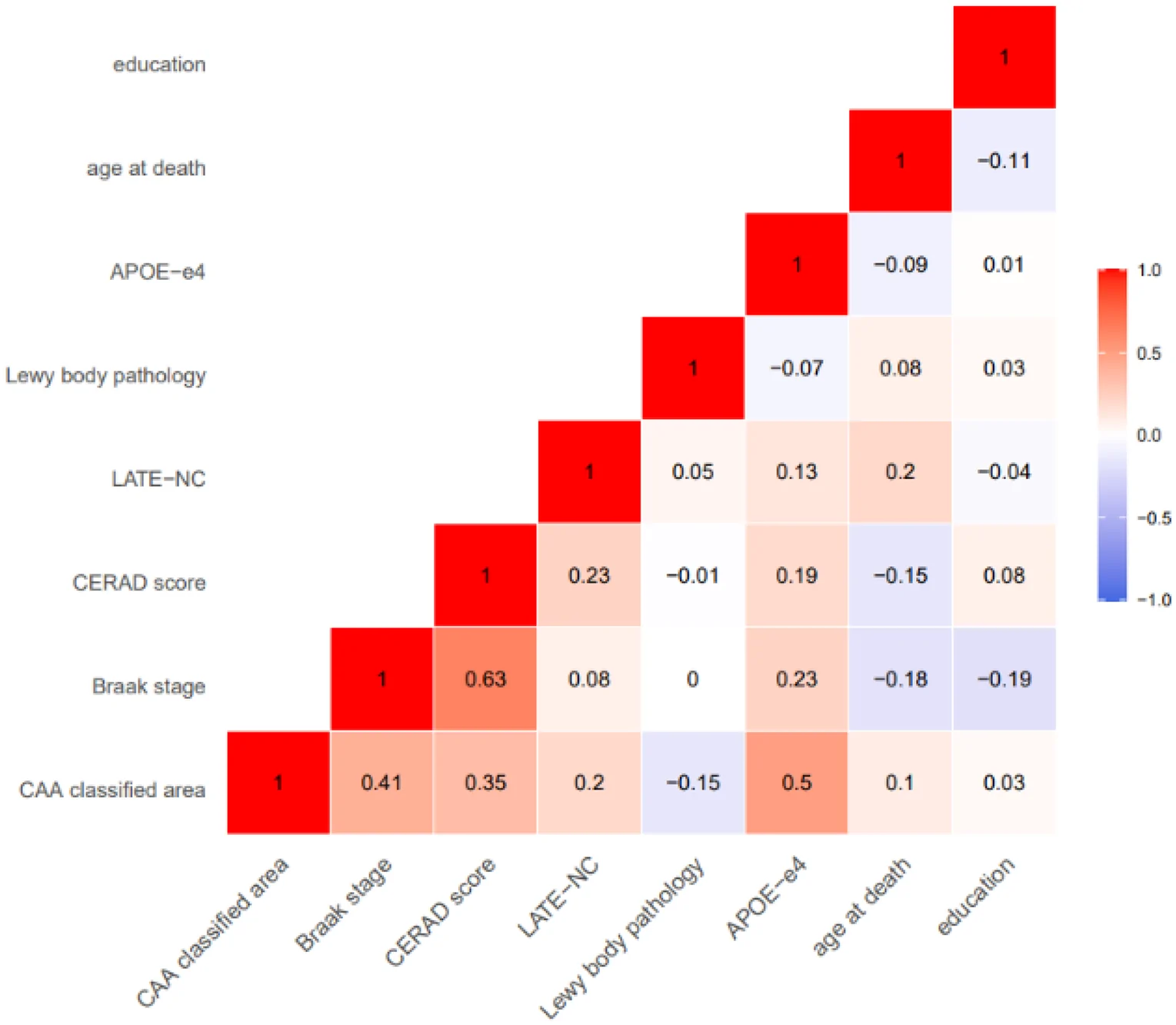
Spearman correlations of digital CAA and β-amyloid plaque measures with neuropathologic variables

**Table 1:** Descriptive of participants (n = 100)

	All participants (N = 100), mean (SD) or N (%)
*Demographics*	
Age at death, years	91.1 (5.82)
Sex, women	76 (76%)
Education, years	16.6 (3.42)
Any APOE-e4 allele*	24/91 (26%)
*Clinical*	
MMSE	22.12 (7.80)
NCI	30 (30%)
MCI	20 (20%)
Dementia	50 (50%)
*Neuropathology*	
Alzheimer's Disease Neuropathologic Change (ADNC)	
No ADNC	6 (6%)
Low likelihood	13 (13%)
Intermediate likelihood 51 (51%)	
High likelihood	30 (30%)
A-score**	
0	6 (6%)
1	8 (8%)
2	32 (32%)
3	53 (54%)
B-Score	
0	0 (0%)
1	10 (10%)
2	54 (54%)
3	36 (36%)
C-Score	
No AD	11 (11%)
Possible	4 (4%)
Probable	47 (47%)
Definite	38 (38%)
LATE-NC	
No LATE-NC	33 (33%)
Stage 1	16 (16%)
Stage 2	15 (15%)
Stage 3	36 (36%)
LB pathology present	29 (29%)

* Genotypes for the APOE were not available for 9 participants.

** A score could not be evaluated for one participant.

**Table 2:** Digital quantitative measures by manual CAA scores

Number of participants	All	Score 0	Score 1	Score 2	Score 3	Score 4
	100	20	20	20	20	20
Number of CAA- positive vessels, median (IQR)	13.00 (2.00–167.50)	0 (0–1)	3 (2–4.75)	11.50 (5–14.80)	152.50 (98.50–229.50)	399 (287–652)
CAA classified area (mm^2^), median (IQR)	0.018 (0.002–0.276)	0 (0.00–0.00)	0.006 (0.002–0.011)	0.014 (0.006–0.030)	0.233 (0.142–0.347)	0.598 (0.447–1.071)
Vascular β-amyloid percent positivity, median (IQR)	32.93 (10.89–50.33)	0 (0.00–0.00)	24.94 (10.37–34.10)	29.95 (21.47–43.41)	39.73 (33.10–52.16)	57.91 (50.05–68.47)
Number of total parenchymal β-amyloid plaques, median (IQR)	1884.50 (325–3625.50)	142 (0.50–1526)	2598.50 (383–3544.50)	1699.50 (713.50–3422)	2089.50 (495–4045)	2937 (1427.50–4157.50)
Plaque classified area (mm^2^), median (IQR)	1884.50 (325.00–3625.50)	0.12 (0.00–0.86)	2.01 (0.36–3.18)	1.69 (0.71–3.02)	1.88 (0.38–3.21)	1.95 (0.54–3.36)

Quantitative metrics are obtained across the entire cortical region of interest.

**Table 3:** Density of β-amyloid perivascular plaques within concentric rings surrounding CAA-positive vessels.

Perivascular plaques/μm^2^ area centered within 0–50-micron distance from vessel, median (IQR)	Score 0	Score 1	Score 2	Score 3	Score 4
	**NA**	**1.27** **(0–2.55)**	**1.27** **(0–7)**	**22.28** **(3.82–49.66)**	**87.22** **(19.74–171.89)**
Perivascular plaques/μm^2^ area centered within 50–100-micron distance from vessel, median (IQR)	NA	0.85 (0–0.85)	1.70 (0.42–2.97)	15.70 (2.76–44.56)	54.75 (29.92–145.15)
Perivascular plaques/μm^2^ area centered within 100–150-micron distance from vessel, median (IQR)	NA	0.00 (0–1.27)	0.76 (0.25–2.16)	13.37 (2.42–24.19)	48.77 (23.05–199.05)

Reported values were calculated by dividing perivascular plaque counts by each concentric ring area (μm^2^) and multiplied by 10^4^.

## Data Availability

Raw data are available by request through the Rush Alzheimer’s Disease Center (RADC) Research Resource Sharing Hub www.radc.rush.edu.
